# Sex Differences in the Vasodilation Mediated by G Protein-Coupled Estrogen Receptor (GPER) in Hypertensive Rats

**DOI:** 10.3389/fphys.2021.659291

**Published:** 2021-07-29

**Authors:** Nathalie Tristão Banhos Delgado, Wender do Nascimento Rouver, Leandro Ceotto Freitas-Lima, Ildernandes Vieira-Alves, Virgínia Soares Lemos, Roger Lyrio dos Santos

**Affiliations:** ^1^Department of Physiological Sciences, Health Sciences Center, Federal University of Espirito Santo, Vitoria, Brazil; ^2^Department of Biophysics, Federal University of São Paulo, São Paulo, Brazil; ^3^Department of Physiology and Biophysics, Federal University of Minas Gerais, Belo Horizonte, Brazil

**Keywords:** GPER, G-1, hypertension, mesenteric resistance arteries, estrogen, vascular reactivity

## Abstract

**Background:**

The protective effect of estrogen on the vasculature cannot be explained only by its action through the receptors ERα and ERβ. G protein-coupled estrogen receptors (GPER)—which are widely distributed throughout the cardiovascular system—may also be involved in this response. However, little is known about GPER actions in hypertension. Therefore, in this study we evaluated the vascular response mediated by GPER using a specific agonist, G-1, in spontaneously hypertensive rats (SHR). We hypothesized that G-1 would induce a relaxing response in resistance mesenteric arteries from SHR of both sexes.

**Methods:**

G-1 concentration-response curves (1 nM-10 μM) were performed in mesenteric arteries from SHR of both sexes (10–12-weeks-old, weighing 180–250 g). The effects of G-1 were evaluated before and after endothelial removal and incubation for 30 min with the inhibitors L-NAME (300 μM) and indomethacin (10 μM) alone or combined with clotrimazole (0.75 μM) or catalase (1,000 units/mL). GPER immunolocalization was also investigated, and vascular hydrogen peroxide (H_2_O_2_) and ROS were evaluated using dichlorofluorescein (DCF) and dihydroethidium (DHE) staining, respectively.

**Results:**

GPER activation promoted a similar relaxing response in resistance mesenteric arteries of female and male hypertensive rats, but with the participation of different endothelial mediators. Males appear to be more dependent on the NO pathway, followed by the H_2_O_2_ pathway, and females on the endothelium and H_2_O_2_ pathway.

**Conclusion:**

These findings show that the GPER agonist G-1 can induce a relaxing response in mesenteric arteries from hypertensive rats of both sexes in a similar way, albeit with differential participation of endothelial mediators. These results contribute to the understanding of GPER activation on resistance mesenteric arteries in essential hypertension.

## Introduction

Cardiovascular disease (CVD) is the leading cause of death worldwide and men are known to be more susceptible to it when compared to premenopausal women (Benjamin et al., 2019). Among risk factors for CVD, hypertension stands out as the most prevalent one (MacMahon et al., 1990; Vasan et al., 2001; Chobanian et al., 2003). Hypertension is a polygenic disease that results from abnormalities in blood pressure control mechanisms, characterized by elevated and sustained blood pressure levels, increased peripheral vascular resistance (Doggrell and Brown, 1998; Tahvanainen et al., 2006), and endothelial dysfunction (Lerman et al., 2019). Epidemiological studies demonstrate that women who are in the pre-menopause period are at a lower risk of developing hypertension when compared to those in post-menopause (Heron, 2010; Benjamin et al., 2019), attributing to sex hormones a protective role in the vascular system (Farhat et al., 1996; Mosca et al., 2007).

Among sex hormones, estrogen has already had its protective effects on vessels well-described. The vascular protection provided by estrogen can be mediated by a direct action on endothelial cells (Haynes et al., 2003), acting as a potent stimulator for endothelial nitric oxide synthase (eNOS) activation or increasing the nitric oxide (NO) bioavailability through various mechanisms, such as (i) reducing the formation of reactive oxygen species (ROS) (Yen et al., 2001); (ii) up-regulating eNOS RNAm expression (Stirone et al., 2003) and (iii) inhibiting the expression of the gp91phox subunit of the NADPH oxidase system, consequently reducing the formation of ROS in endothelial cells (Wagner et al., 2001). In addition, the beneficial effects of estrogen appear to be, in part, due to its modulatory effects in the production of prostanoids (PNs) (Hermenegildo et al., 2005), for instance, in rat cerebral blood vessels (Ospina et al., 2002), as well as in human umbilical vein endothelial cells through ERα (Sobrino et al., 2010) and in the carotid arteries of mice via ERα36 (Costa et al., 2019). Although two types of estrogen receptors have already been described, ERα (Soloff and Szego, 1969) and ERβ (Kuiper et al., 1996), the protective effect of estrogen on the vasculature (Reslan and Khalil, 2012) cannot be explained only by these receptors (Revankar et al., 2005).

In the mid-90s, orphan G-protein-coupled receptors (GPCR) were cloned from human lymphoblastic B cells (Owman et al., 1996). These receptors were initially designated as GPR30, due to the consecutive number of orphaned GPCR reported in the literature. Takada et al. (1997) reported on the role played by these receptors in human umbilical vein endothelial cells exposed to shear stress, while working on cloning cDNAs encoding GPCRs in these cells. Theirs was the first study to demonstrate the participation of GPCRs in the response of vessels to shear stress, although no specific ligand was described then. The first indications that these GPR30s may be related to estrogen responsiveness were described by Carmeci et al., 1997. In 2005, Thomas et al. (2005) established the concept that GPR30 is an estrogen receptor, leading to it being described as G protein-coupled estrogen receptor (GPER). It is located on and in the cell membrane—though predominantly in the endoplasmic reticulum membrane, being involved in extranuclear mechanisms (Revankar et al., 2005; Prossnitz et al., 2008; Gaudet et al., 2015).

GPER is widely distributed throughout the cardiovascular system, suggesting an important physiological role on its regulation (Deschamps and Murphy, 2009; Haas et al., 2009; Jessup et al., 2010). Peixoto et al. (2017, 2018) reported sex differences regarding GPER expression in resistance mesenteric arteries of normotensive rats. Higher expression was found in males, although females had higher levels of GPER in the endothelium than in vascular smooth muscle (VSM). GPER activation provides protective effects, such as maintaining blood glucose (Mårtensson et al., 2009), anti-atherogenic effects (Meyer et al., 2014), and relaxation of resistance mesenteric arteries (Deschamps and Murphy, 2009; Peixoto et al., 2017). According to previous results from our group using normotensive models, G-1 induced vascular relaxation in mesenteric resistance arteries, what was not influenced by sex (Peixoto et al., 2017, 2018). Also, this response was, at least in part, endothelium- dependent, especially in females (Peixoto et al., 2017). Although GPER expression was significantly higher in male rats than in females (Peixoto et al., 2018), the activation of the PI3K-Akt-eNOS pathway and potassium channels was similar in both sexes (Peixoto et al., 2017). Despite this evidence in normotensive animals, the vascular role of GPER in hypertension remains uncertain.

Based on the evidence described above, we hypothesize that the GPER agonist, G-1, induces the relaxation of resistance mesenteric arterial segments in hypertensive rats of both sexes. Therefore, the objective of our work was to evaluate the role of GPER activation in resistance mesenteric arteries of spontaneously hypertensive rats (SHR) of both sexes, as well as to identify possible endothelial mediators involved in this response.

## Materials and Methods

### Experimental Animals

For this study, we used 12-weeks-old male SHR (262 ± 9 g of body weight) and female SHR (150 ± 5 g of body weight), provided by the animal facility of the Health Sciences Center of the Federal University of Espirito Santo. All procedures were conducted in accordance with the recommendations of the Brazilian Guidelines for the Care and Use of animals for Scientific and Didactic Purposes and the Guidelines for the Practice of Euthanasia (CONCEA-MCT, 2013), having been approved by the Animal Ethics Committee from the Federal University of Espirito Santo (No. #48/2016). The animals were maintained in group-housing under controlled conditions of temperature (22–24°C) and humidity (40–60%), with a 12/12-h light-darkness cycle, with water and food *ad libitum*. Blood pressure was measured by tail plethysmography before euthanasia.

### Vaginal Smears

The females’ oestrous cycles were monitored using vaginal smears. Vaginal fluid was collected daily from each animal between 08:00 and 09:00 h. Vaginal epithelial cells were examined under an optical microscope as described by Marcondes et al. (2002), for the identification of the different stages of the oestrous cycle. We chose to use rats in the dioestrus phase in order to avoid possible interference in the response, since this phase is characterized by presenting lower levels of estrogen, thus avoiding possible competition between the hormone and the agonist G-1 for GPER. Following a similar schedule, male rats underwent the same handling procedure daily, to reproduce the possible stress suffered by the females.

### Vascular Reactivity

Vascular reactivity of the mesenteric arteries was assessed through a resistance myograph system (620 M; Danish Myo Technology, Aarhus, Denmark). The protocols were performed according to a method previously described by Mulvany and Halpern (1977). In order to prevent interference with the sustained phase of the contractile response, the rats were euthanized by decapitation without anesthesia (Hatano et al., 1989). Third-order mesenteric arteries were isolated, dissected from the adjacent tissue, cut into 2 mm rings and mounted between 2 tungsten threads (40 μm in diameter) inside chambers filled with Krebs solution containing: NaCl, 119 mM; KCl, 4.7 mM; KH_2_PO_4_, 0.5 mM; NaHCO_3_, 13,4 mM; MgSO_4_.7H_2_O, 1.17 mM; CaCl_2_.2H_2_O, 2.5 mM; and glucose, 5.5 mM, kept at 37 °C and aired with carbogenic mixture (95% O_2_ e 5% CO_2_). The rings were gradually stretched until their internal diameters corresponded to a transmural pressure of 100 mmHg and the internal circumference (IC1) was then normalized to a set fraction of the internal circumference (IC100). Thus, IC1 was calculated by multiplying IC100 by 0.9. Endothelial viability and integrity were assessed by administration of acetylcholine (ACh, 10 μM) in rings previously contracted by phenylephrine (PE, 3 μM). The endothelium was considered viable when the relaxation response observed was ≥ 80%. Following mechanical removal of the endothelium, the vessels were rated as endothelium-free when the ACh-induced relaxation was < 10%.

Concentration-response curves were plotted following the cumulative addition (1 nM-10 μM) of G-1 (1-[4-(6-bromobenzo [1,3]dioxol-5yl)-3a,4,5,9b-tetrahydro-3Hcyclopenta-[c]quinolin-8-yl]-ethanone; Cayman Chemical, MI, United States), a non-steroidal, high -affinity GPER agonist (Bologa et al., 2006), following the previous induction of contraction with PE (3 μM). The G-1 vasodilator effect was investigated in the presence of inhibitors: N^ω^-nitro-L-arginine methyl ester (L-NAME, NOS inhibitor, 300 μM; Sigma, St. Louis, MO, United States), a combination of L-NAME (300 μM) and indomethacin (INDO, cyclooxygenase—COX—inhibitor, 10 μM; Sigma, St. Louis, MO, United States), L-NAME, INDO and clotrimazole (CLOT, cytochrome P450—CYP—inhibitor, 0.75 μM Sigma, St. Louis, MO—United States) and L-NAME, INDO and an enzyme that specifically decomposes hydrogen peroxide (H_2_O_2_) (Catalase, 1,000 units/mL). Samples were incubated for 30 min. The percent relaxation was determined using a LabChart 8 data acquisition system (AD Instruments Pty Ltd., New South Wales, Australia).

### Immunofluorescence of GPER

Immunofluorescence of GPER was performed according to the protocol described by Aires et al. (2013), with some modifications. Briefly, slides with cross-sections (10 μm) of mesenteric arteries were fixed in 4% PFA for 15 min and washed with 1% BSA (1% BSA + 0.3% Triton X –100 in PBS). Blocking was performed with a 3% BSA solution diluted in PBS 1x-0.3% Triton-x for 30 min. The slides were then washed with BSA 1% and incubated in a humid chamber at 4°C overnight with the primary antibodies: rabbit anti-GPER (1: 400, Abcam, #ab39742) and sheep anti-VWF (1:300, Abcam, #ab11713). Next, incubation was performed for 1 h at room temperature with secondary antibodies: goat anti-rabbit Alexa Fluor 555 (1:300, Invitrogen, #35552) and donkey anti-sheep Alexa Fluor 488 (1:300, Abcam, #ab150178). Nuclear staining was obtained by incubation with 4,6-diamidino-2-phenylindole (DAPI, 1:300). Negative controls were obtained using blocking solution (3% BSA) without adding the primary antibody. Image acquisition was performed in an Apotome microscope (Zeiss, Germany) with 488 and 555 nm filters. Fluorescence analysis was performed using Fiji software^®^ version 1.53 (National Institute of Health, United States). The intensity of total vascular fluorescence of anti-GPER and its colocalization with anti-VWF (merge between 488 and 555 filters) was measured in two rings of each animal, normalized by the analyzed area. Results were expressed as arbitrary units (Caracuel et al., 2012).

### Evaluation of the *in situ* Production of Hydrogen Peroxide (H_2_O_2_) and Reactive Oxygen Species (ROS)

The *in situ* production of H_2_O_2_ and ROS was evaluated with the probes 2’ diacetate, 7’ dichlorodihydrofluorescein (H2DCF-DA; Cayman, # 05655259) and Dihydroethidium (DHE, Cayman, # 056381010), respectively, following previously published methods, with some modifications (Carrillo-Sepulveda et al., 2015; Couto et al., 2015; Loh et al., 2015; Silva et al., 2016). For this purpose, third-order mesenteric artery segments were isolated, included in freezing medium (Tissue-Tek^®^ OCT^TM^, Sakura^®^, United States), and cut into 8 μM transverse segments (Cryostat et al., 1850, Leica). To elucidate the participation of G-1 in the production of H_2_O_2_ and ROS, evaluations took place in three different moments: (I) investigation of basal production; (II) analysis of H_2_O_2_ and ROS production after stimulation with GPER agonist G-1 (10 μM), and (III) qualitative analysis of the production of these substances after stimulation with G-1 (10 μM) combined with the use of catalase (1,000 units / ml) or the ROS scavenger Tiron (10 μM). For this, on the day of the experiment, slides containing the sections were thawed at room temperature from −80°C storage, equilibrated in Phosphate-buffered saline (PBS) for 15 min, and incubated with H2DCF-DA (10 μM) or DHE (5 μM) for 30 min in a humid chamber and protected from light for further 30 min. After this period, the sections were incubated with G-1, catalase, and/or Tiron for 30 min, under the same conditions described for incubation with the probes. The slides used to assess basal ROS production received the same amount of PBS and the negative control slides did not receive the fluorescent probes. Digital images were acquired with a Zeiss fluorescence microscope (Zeiss, 174 Oberkochen, Germany) using 488 and 546 nm excitation and a 20x objective. Fluorescence intensity was measured using ImageProPlus software (version 4.0) and expressed in arbitrary units (A.U.).

### Statistical Analysis

Data analysis was performed with GraphPad Prism 6 (GraphPad Software, La Jolla, CA, United States). All data are expressed as mean ± standard error of the mean (SEM). Data normality was evaluated by the Shapiro-Wilk test. Once normality was confirmed, comparisons were performed through two-way analysis of variance (two-way ANOVA) followed by Sidak *post hoc* test. The area under the curves (AUC) was evaluated through one-way analysis of variance (one-way ANOVA) followed by Tukey *post hoc* test. The immunofluorescence of GPER, Mann-Whitney test was used for the analysis. The significance level was set at *P* < 0.05.

## Results

### Vascular Reactivity

**FIGURE 1:**
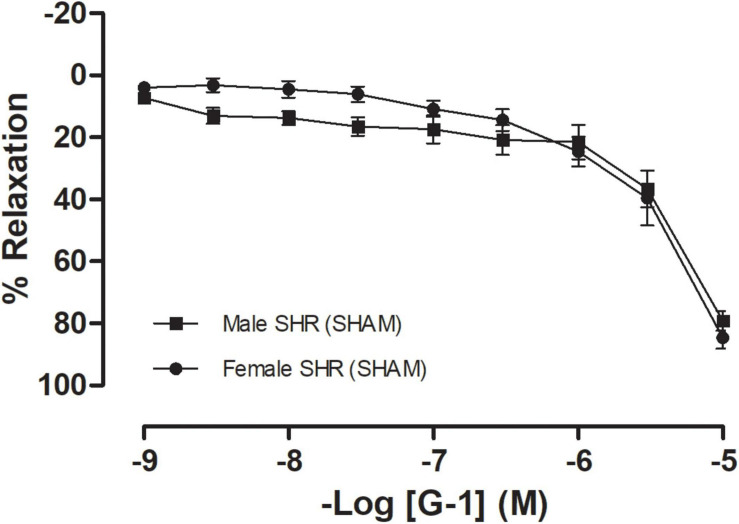
Concentration-response curves for the selective GPER agonist G-1 (1 nM-10 μM) in mesenteric resistance arteries are similar in both sexes. Female SHR (*n* = 10) are represented by circles and male SHR (*n* = 13) by squares. Reactivity protocols were performed in females during the diestrus cycle. Values are expressed as mean ± SEM. The curve was analyzed point-by-point through two-way ANOVA, followed by the Sidak *post hoc* test.

To confirm hypertension in SHR, blood pressure was assessed non-invasively by tail plethysmography. We observed that these animals have increased blood pressure values, with significant differences having been detected between sexes regarding systolic blood pressure (Males: 215 ± 5 and Females 182 ± 4 mmHg), diastolic blood pressure (Males: 178 ± 7 and Females 143 ± 3 mmHg), and heart rate (Males: 362 ± 15 and Females 343 ± 2 bpm). The GPER-mediated vascular response was assessed using mesenteric resistance arteries from both female and male SHR. G-1, a GPER agonist, induced a concentration-dependent relaxation on mesenteric resistance arteries from both females (79 ± 3%) and males (83 ± 3%), with no significant differences having been observed between sexes (Figure 1).

**FIGURE 2:**
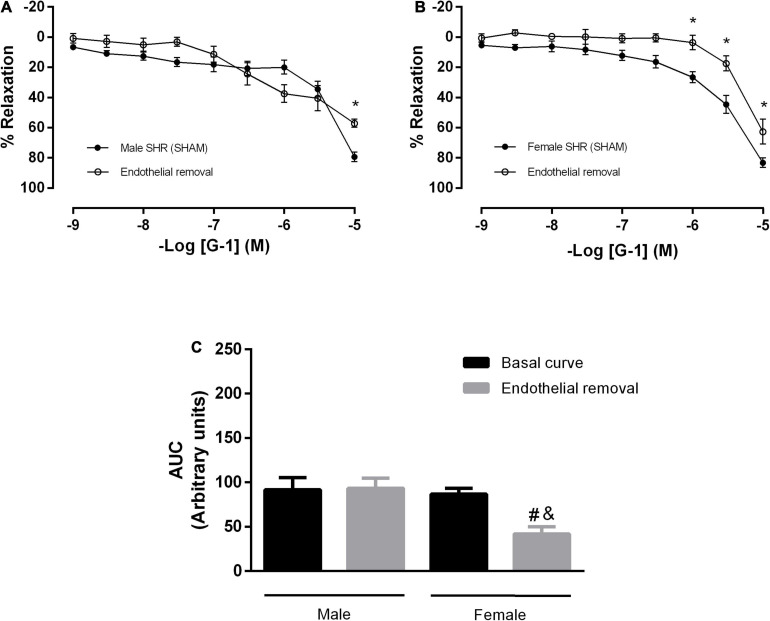
The vasodilator response induced by G-1 is partially dependent on the endothelium in female SHR. Effect of endothelial removal on the relaxation response in **(A)** Male (*n* = 5) and **(B)** Female (*n* = 6) SHR groups. **(C)** Area under the curve (AUC) before and after endothelial removal. Values are expressed as mean ± SEM. *P < 0.05 compared to the same dose in the control curve, ^#^*P* < 0.05 compared to the AUC of the Female SHR control curve and ^&^*P* < 0.05 compared to the AUC of the Male SHR group after endothelial removal. The curve analysis was carried out point-by-point through two-way ANOVA, followed by the Sidak *post hoc* test. AUCs were evaluated through one-way ANOVA followed by Tukey *post hoc* test.

Next, we verified the participation of the endothelium in the GPER-mediated relaxation in resistance mesenteric arteries from SHR. After endothelial removal (Figures 2A,B), we observed a reduction in the vasodilator response induced by G-1 in both sexes (male: 79 ± 3 to 57 ± 3% and female: 83 ± 3 to 62 ± 8%), but a significant reduction in AUC was observed only in the female group (86 ± 6 to 41 ± 8 A.U.), as seen in the Figure 2C.

**FIGURE 3:**
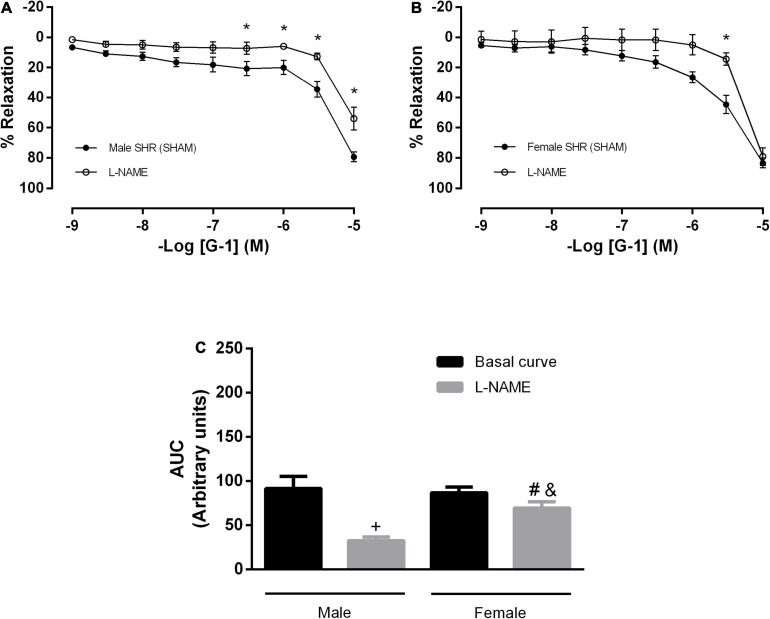
The vasodilator response induced by G-1 is partially dependent on the NO pathway in both sexes, but with a greater participation in male SHR. Effect of non-selective nitric oxide synthase inhibition with N^ω^-nitro-L-arginine methyl ester (L-NAME, 300 μM) in **(A)** Male (*n* = 8) and **(B)** Female (*n* = 6) SHR groups. **(C)** Area under the curve (AUC) before and after 30 min with L-NAME incubation. Values are expressed as mean ± SEM. *P < 0.05 compared to the same dose in the control curve, ^+^*P* < 0.05 compared to the AUC of the Male SHR control curve, ^#^*P* < 0.05 compared to the AUC of the Female SHR control curve and ^&^*P* < 0.05 compared to the AUC of the Male SHR group after L-NAME incubation. The curve analysis was carried out point-by-point through two-way ANOVA, followed by the Sidak *post hoc* test. AUCs were evaluated through one-way ANOVA followed by Tukey *post hoc* test.

We then attempted to identify which endothelial mediators could be involved in this response. However, the impairment detected in the NO pathway was higher in males than in females, as the former had a lower relaxation response after L-NAME incubation (males: 79 ± 3 to 54 ± 7 and females: 83 ± 3 to 79 ± 5%) (Figures 3A,B). These results were confirmed when the area under the curves was compared (male: 91 ± 13 to 32 ± 4 and female: 86 ± 6 to 69 ± 6 A.U.) (Figure 3C).

**FIGURE 4:**
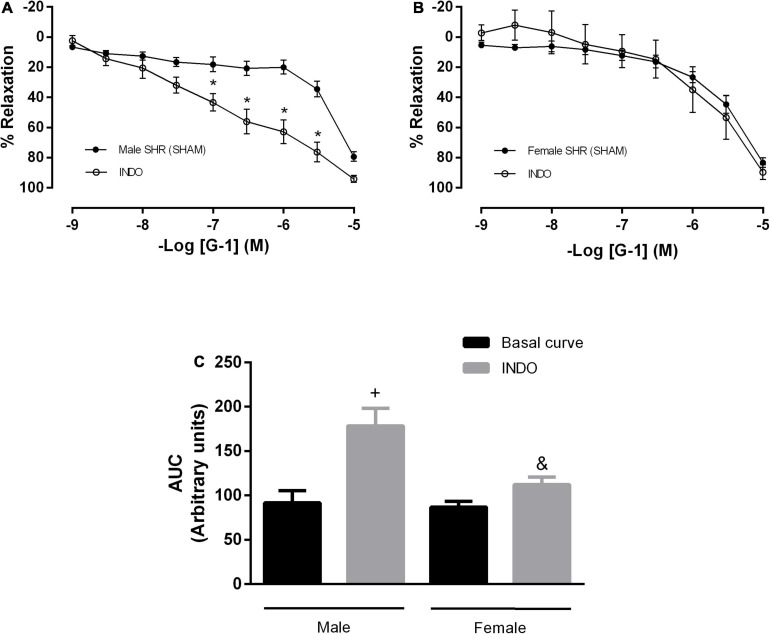
The prostanoids pathway does not participate in the vasodilator response induced by G-1. However, nonspecific inhibition of cyclooxygenase potentiated this response in the Male SHR group. Effect of non-selective cyclooxygenase inhibition with indomethacin (INDO, 10 μM) in **(A)** Male (*n* = 8) and **(B)** Female (*n* = 7) SHR groups. **(C)** Area under the curve (AUC) before and after INDO incubation. Values are expressed as the mean ± SEM. *P < 0.05 compared to the same dose in the control curve, ^+^*P* < 0.05 compared to the AUC of the Male SHR control curve and ^&^*P* < 0.05 compared to the AUC of the Male SHR group after INDO incubation. The curve analysis was carried out point-by-point through two-way ANOVA, followed by the Sidak *post hoc* test. AUCs were evaluated through one-way ANOVA followed by Tukey *post hoc* test.

The second pathway of mediators studied was the PNs pathway. We observed that after indomethacin incubation, males showed no impairment in the maximum response, although there was an increase in the AUC parameter (91 ± 13 to 178 ± 20 A.U.) (Figures 4A,C). In females, there was no difference in the vasodilator response induced by G-1 nor in the AUC (87 ± 6 to 112 ± 8 A.U.) (Figures 4B,C).

**FIGURE 5:**
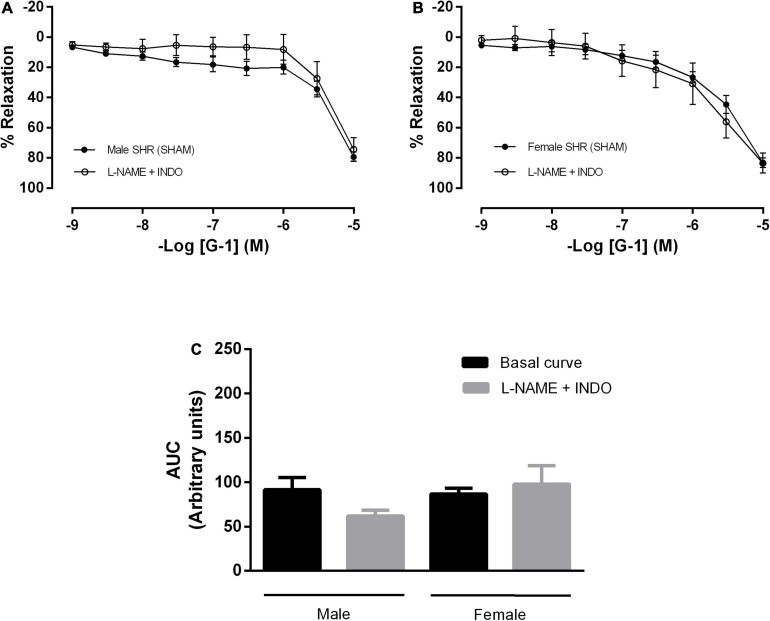
Conjugated inhibition of the NO and prostanoids pathways, without changes in the vasodilator response induced by G-1, indicates the participation of endothelium-dependent hyperpolarization in both sexes. Effect of non-selective nitric oxide synthase inhibition with N^ω^-nitro-L-arginine methyl ester (L-NAME, 300 μM) and cyclooxygenase inhibition with indomethacin (INDO, 10 μM) in **(A)** Male (*n* = 8) and **(B)** Female (*n* = 7) SHR groups. **(C)** Area under the curve (AUC) before and after L-NAME and INDO incubation. Values are expressed as mean ± SEM. The curve analysis was carried out point-by-point through two-way ANOVA, followed by the Sidak *post hoc* test. AUCs were evaluated through one-way ANOVA followed by Tukey *post hoc* test.

**FIGURE 6:**
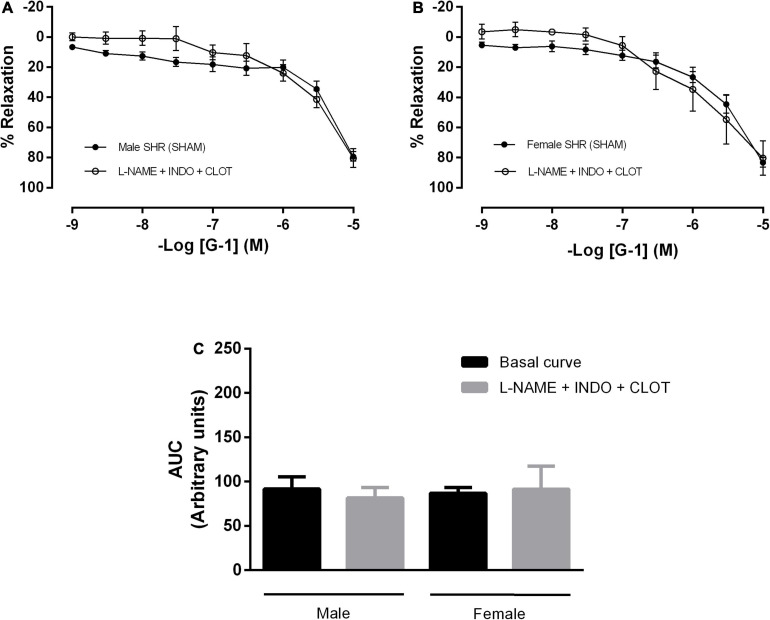
The cytochrome P450 (CYP) pathway, one of the pathways of endothelium-dependent hyperpolarization, does not participate in the vasodilator response induced by G-1 in either sex. Effect of non-selective nitric oxide synthase inhibition with N^ω^-nitro-L-arginine methyl ester (L-NAME, 300 μM), cyclooxygenase inhibition with indomethacin (INDO, 10 μM) and cytochrome P450 (CYP) inhibition (Clotrimazole, 0.75 μM) in **(A)** Male (*n* = 5) and **(B)** Female (*n* = 5) SHR groups. **(C)** Area under the curve (AUC) before and after L-NAME, INDO, and Clotrimazole incubation. Values are expressed as mean ± SEM. The curve analysis was carried out point-by-point through two-way ANOVA, followed by the Sidak *post hoc* test. AUCs were evaluated through one-way ANOVA followed by Tukey *post hoc* test.

In addition, mesenteric resistance arteries were concomitantly incubated with inhibitors for NO and PNs formation, what did not alter the response mediated by GPER in either sex (Figures 5A–C). There were also no differences in the G-1 vasodilator response upon the inhibition of NO, PNs and epoxyeicosatrienoic acids (EETs), arachidonic acid metabolites produced by the activity of the cytochrome P450 enzyme (Figures 6A–C).

**FIGURE 7:**
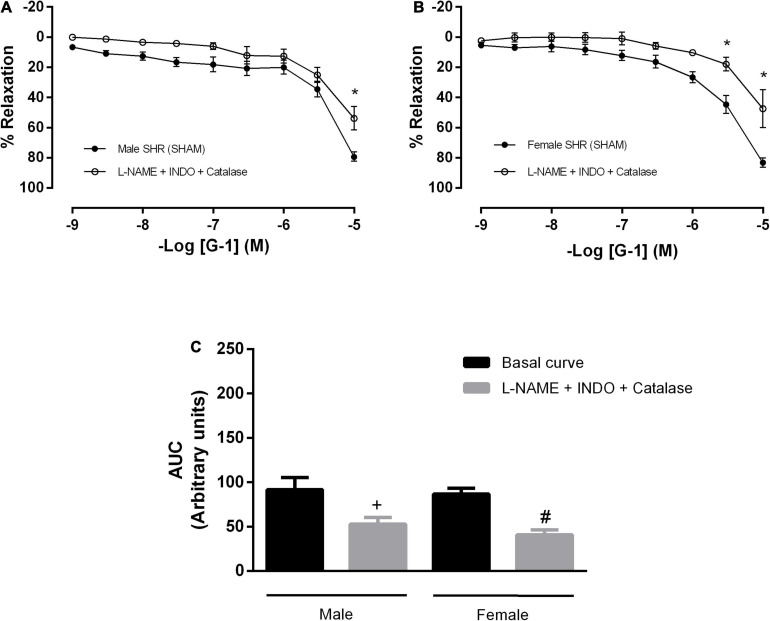
Hydrogen peroxide (H_2_O_2_), another pathway of endothelium-dependent hyperpolarization, participates in the vasodilator response induced by G-1 in both sexes. Effect of non-selective nitric oxide synthase inhibition with N^ω^-nitro-L-arginine methyl ester (L-NAME, 300 μM), cyclooxygenase inhibition with indomethacin (INDO, 10 μM) and an enzyme that specifically decomposes H_2_O_2_ (Catalase, 1000 units/mL) in **(A)** Male (*n* = 6) and **(B)** Female (*n* = 4) SHR groups. **(C)** Area under the curve (AUC) before and after L-NAME, INDO, and catalase incubation. Values are expressed as mean ± SEM. *P < 0.05 compared to the same dose in the control curve, ^+^*P* < 0.05 compared to the AUC of the Male SHR control curve and ^#^*P* < 0.05 compared to the AUC of the Female SHR control curve. The curve analysis was carried out point-by-point through two-way ANOVA, followed by the Sidak *post hoc* test. AUCs were evaluated through one-way ANOVA followed by Tukey *post hoc* test.

In order to verify the participation of H_2_O_2_ in the vasodilator response evoked by G-1 in mesenteric arteries of SHR, we use an enzyme that degrades H_2_O_2_ (catalase) in association with inhibitors of NO and PNs formation. As a result, we observed an impaired vasodilator response in both males and females (males: 79 ± 3 to 53 ± 7 and females: 83 ± 3 to 47 ± 12%) (Figures 7A–C).

**FIGURE 8:**
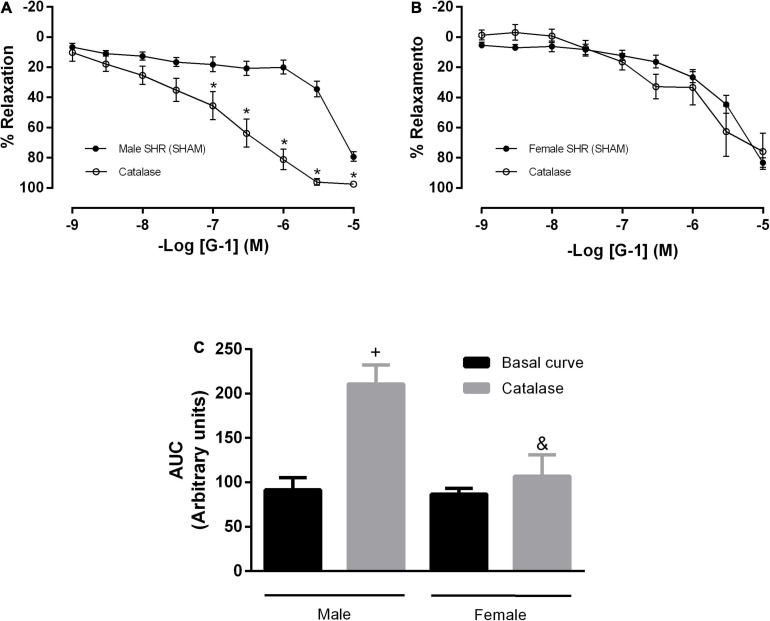
Concentration-response curves of the selective GPER agonist G-1 (1 nM-10 μM) in mesenteric resistance arteries from both sexes. Isolated effect of an enzyme that specifically de composes H_2_O_2_ (Catalase, 1,000 units/mL) in **(A)** Male (*n* = 7) and **(B)** Female (*n* = 5) SHR groups. **(C)** Area under the curve (AUC) before and after catalase incubation. Values are expressed as the mean ± SEM. *P < 0.05 compared to the same dose in the control curve, ^+^*P* < 0.05 compared to the AUC of the Male SHR control curve and ^&^*P* < 0.05 compared to the AUC after catalase incubation of Male SHR group. The curve analysis was carried out point-by-point through two-way ANOVA, followed by the Sidak *post hoc* test. AUCs were evaluated through one-way ANOVA followed by Tukey *post hoc* test.

Silva et al. (2017) observed that H_2_O_2_ has an important role in the cross-talk between NOS and COX, depending on its concentration. Therefore, we analyzed the participation of H_2_O_2_ in this response in the presence of the NO and PNs pathways. Upon isolated incubation with catalase, we observed an increase in the vasodilator response induced by G-1 (Figure 8A) and in the AUC (91 ± 13 to 210 ± 21) (Figure 8C) in SHR males only. These parameters remained unchanged in the female SHR group (Figures 8B,C; AUC: 86 ± 6 to 106 ± 23).

### Immunolocalization of GPER

**FIGURE 9:**
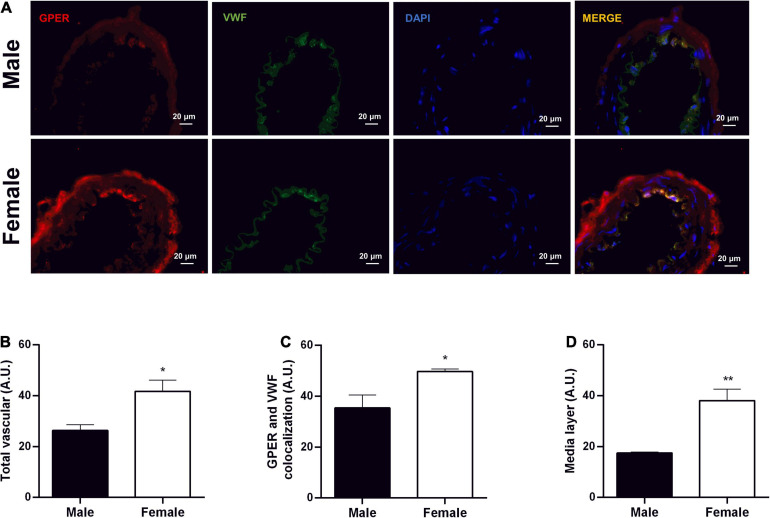
Greater fluorescence intensity of GPER in mesenteric resistance arteries of female SHR. Immunolocalization for GPER **(A)** and fluorescence analysis of total vascular area **(B)**, endothelium area **(C)**, and vascular smooth muscle area **(D)** in Male (*n* = 5) and Female (*n* = 4) SHR groups. Scale bar = 20 μm. Values are expressed as mean ± SEM. ^∗^*P* < 0.05 and ^∗∗^*P* < 0.01 compared with Male SHR group. Mann-Whitney test was used for the analysis.

Immunofluorescence analysis was performed on resistance mesenteric arteries to identify the presence of GPER in SHR of both sexes. The results indicate higher fluorescence levels in the total vascular area (males: 26 ± 2 and females: 41 ± 4 A.U.), endothelial area (males: 35 ± 5 and females: 49 ± 1 A.U.), and medial layer (males:17.5 ± 0.3 and females: 38 ± 4.5 A.U.) of females compared with males (Figures 9A–D).

### Evaluation of the *in situ* Production of Hydrogen Peroxide (H_2_O_2_) and Reactive Oxygen Species (ROS)

**FIGURE 10:**
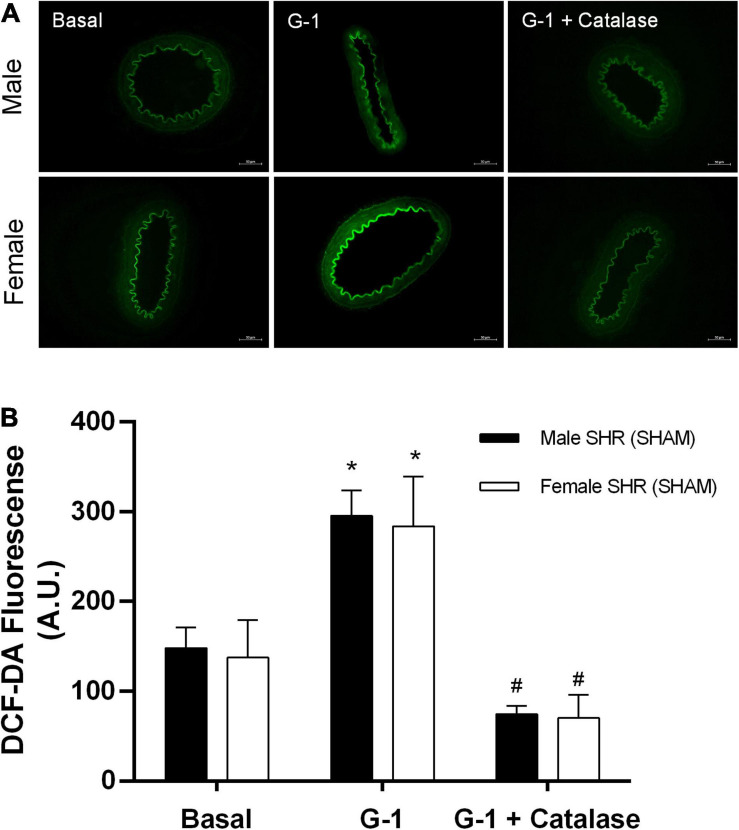
**(A)** Fluorescence microscopy analysis emitted by H2DCF-DA in mesenteric resistance arteries from SHR. Protocols were performed in the absence and presence of G-1 (10 μM) and catalase (1,000 units / mL). **(B)** Fluorescence analysis produced in Male (*n* = 5) and Female (*n* = 6) groups. Scale bar = 50 μm. Values are expressed as mean ± SEM. *P < 0.05 compared with the basal condition of the same group and ^#^*P* < 0.05 compared with G-1 stimulation of the same group. Two-way ANOVA was used for the analysis, followed by the Sidak *post hoc* test.

**FIGURE 11:**
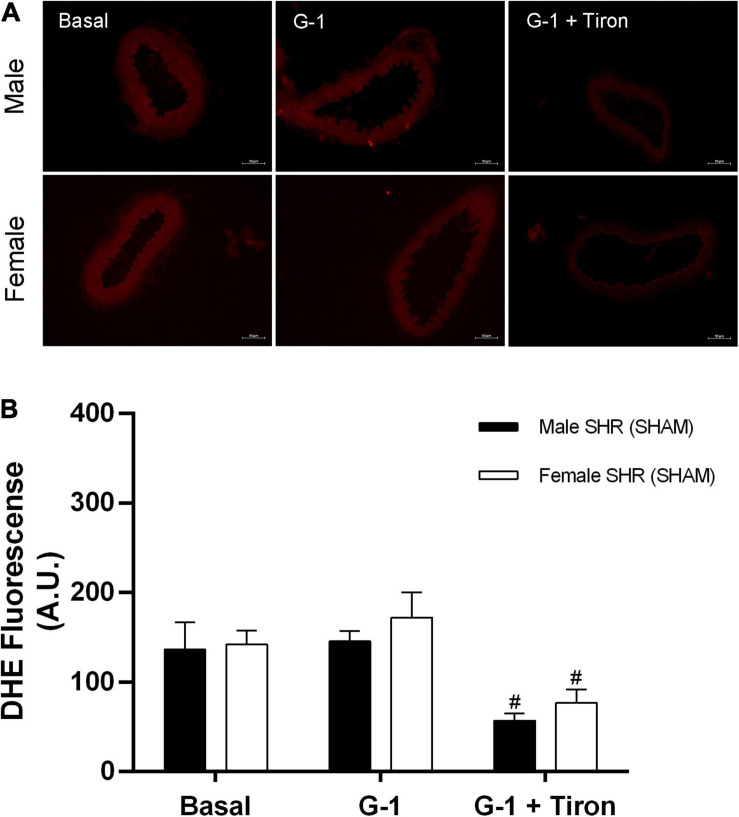
**(A)** Fluorescence microscopy analysis emitted by DHE in mesenteric resistance arteries from SHR. Protocols were performed in the absence and presence of G-1 (10 μM) and Tiron (10 μM), an ROS scavenger. **(B)** Fluorescence analysis produced in Male (*n* = 5) and Female (*n* = 6) groups. Scale bar = 50 μm. Values are expressed as mean ± SEM. ^#^*P* < 0.05 compared with G-1 stimulation of the same group. Two-way ANOVA was used for the analysis, followed by the Sidak *post hoc* test.

Fluorescence analysis for H_2_O_2_ in resistance mesenteric arteries of SHR indicates no differences between sexes (males: 148 ± 23 and females: 137 ± 40 A.U.) in basal conditions, although G-1 can stimulate the production of this compound in both sexes (males: 295 ± 20 and females: 283 ± 50 A.U.). In addition, as a negative control, we used catalase, an enzyme that degrades H_2_O_2_. The presence of catalase reduced the fluorescence intensity related to H_2_O_2_ in both sexes (males: 74 ± 9 and females: 70 ± 24 A.U.) (Figure 10). In relation to the fluorescence analysis for ROS, we also found no differences between males and females (males: 137 ± 30 and females: 142 ± 15 A.U.) in basal conditions, and that G-1 did not stimulate their production in either sex (males: 145 ± 11 or females: 172 ± 28 A.U.). As a negative control, we used the ROS scavenger Tiron, which, as expected, reduced the fluorescence intensity of ROS in both sexes (males: 57 ± 8 and females: 77 ± 14 A.U.) (Figure 11).

## Discussion

The results of this study demonstrated that GPER activation promotes relaxation in mesenteric resistance arteries from both male and female SHR, in a partially endothelium-dependent manner. Among the endothelial mediators evaluated, we observed that the NO pathway is predominant in males and the H_2_O_2_ pathway in females. Thus, despite there being no sex differences in relaxation induced by G-1, the mechanisms involved in this response are different in each sex.

The relaxation response induced by G-1 has also been detected in other arterial segments, as well as other species, such as pig coronaries (Yu et al., 2014), mesenteric resistance arteries of intact Lewis rats (Lindsey et al., 2014), rat aortic segments (Jang et al., 2013) and rat cerebral artery segments (Murata et al., 2013). This same response was also observed in clinical studies (Arefin et al., 2014) and in a recent study in pig coronary arteries (Yu et al., 2017, 2018), as well as in molecular studies conducted with human endothelial cells and knockout mice (Fredette et al., 2018).

As to our observation that the relaxation induced by G-1 was alike in both sexes, it agrees with the results reported by Peixoto et al. (2017), who demonstrated that GPER mediates vasorelaxation effects in resistance mesenteric arteries of Wistar rats without sex differences. Broughton et al. (2010) also reported a relaxing response in rat carotid arteries with no difference between males and females. On the other hand, another study from our group demonstrated that the relaxation induced by G-1 in the coronary vascular bed of rats was greater in females than in males, an effect explained by the greater presence of ROS in males (Debortoli et al., 2017). Taken together, these results indicate that the activation of GPER in different vascular beds has yet to be fully elucidated.

We have also observed that the relaxing response induced by G-1 is partially dependent on the endothelium, for after endothelial removal relaxation was impaired in both sexes. Similar results were found in coronary arteries (Yu et al., 2011) and in mesenteric arteries (Peixoto et al., 2017). Several studies have demonstrated endothelium-dependent effects of GPER, such as NO-dependent vasodilation (Fredette et al., 2018) and increased expression of eNOS (Arefin et al., 2014). The endothelium plays an important role in maintaining vascular tone by synthesizing and releasing several endothelium−derived relaxing factors (EDRF), i.e., NO, PNs and endothelium-dependent hyperpolarization (EDH) (Félétou et al., 2011; Leung and Vanhoutte, 2017; Vanhoutte et al., 2017). Notwithstanding, activation of GPER can affect VSM as well (Murata et al., 2013). Indeed, Lindsey et al. (2014) observed relaxation through both an endothelial-dependent mechanism and a direct action on VSM in mesenteric resistance arteries of Lewis female rats following GPER activation. In VSM, this activation may couple to that of the G-protein αs subunit, to activate adenylyl cyclase (AC), increase cAMP, and trigger protein kinases (PKA, PKG) to phosphorylate proteins involved in VSM contractility, for instance, effector proteins that decrease the contractile state of VSM. In addition, activation of GPER might also antagonize changes in intracellular calcium evoked by vasoconstrictor agonists and can have direct effects on calcium mobilization, lowering blood pressure in normotensive rats (Haas et al., 2009). However, the objective of this study was to elucidate the endothelial pathways involved in the relaxation mediated by GPER, that is, which endothelial mediators would be involved in this response. First, we tested the NO pathway after incubation with a non-specific NOS inhibitor. We observed a decrease in the vasodilator response, what was reflected by the AUC, though only in males. Although a strong participation of NO in the relaxation induced by G-1 has been previously reported in both sexes (Broughton et al., 2010; Lindsey et al., 2014; Peixoto et al., 2017), in hypertension the involvement of the NO pathway appears to be sex-dependent.

The double inhibition with L-NAME plus indomethacin did not impair the relaxation induced by G-1 in either sex, suggesting that EDH might be involved in this response. We emphasize that, in resistance arteries, EDH pathways play an important role in the regulation of peripheral vascular resistance (Shimokawa et al., 1996; Ohashi et al., 2012) and consequently on blood pressure values. Therefore, clarifications regarding EDH endothelial mediators and their role in blood pressure control mechanisms are important. Within this context, it has been demonstrated that NO-mediated responses are dominant in conductance arteries, while EDH would become more important as the caliber of the arteries reduces (Shimokawa et al., 1996; de Wit and Wolfle, 2007). NO-mediated relaxation is easily impaired, whereas EDH-mediated responses are generally preserved or even enhanced to maintain vascular homeostasis (Kobuchi et al., 2015). Thus, EDH is regarded as a backup system for NO-mediated responses in the maintenance of tissue perfusion. Therefore, elucidating the role of EDH in these arteries is extremely important to understand the mechanisms involved in hypertension (Mori et al., 2006).

EDH was first described in 1988 by Taylor and Weston, who demonstrated that the endothelium was capable of synthesizing a diffusible substance that promoted hyperpolarization of VSM (Taylor and Weston, 1988). EDHs include C-type natriuretic peptide (Chauhan et al., 2003), epoxyeicosatrienoic acids (EETs) (Fissithaler et al., 1999; Fleming, 2000), hydrogen sulfide (H_2_S) (Zhao et al., 2001), potassium ions (K^+^) (Edwards et al., 1998), and electrical communication through myoendothelial gap junctions (Kühberger et al., 1994). These mechanisms activate different families of K^+^ channels, leading to hyperpolarization of VSM cells, contributing to mechanisms that promote relaxation (Shimokawa and Morikawa, 2005; Félétou and Vanhoutte, 2009). First, we tested the contribution of the cytochrome P450-mediated response and observed that there was no participation of this pathway in either males or females. Nevertheless, several studies have pointed to the CYP pathway as an EDH (Triggle et al., 2012) in coronary arteries (Fleming et al., 2001). In mesenteric microvessels from insulin-resistant obese Zucker rats, down-regulation of CYP epoxygenases was found to be associated with impaired vasodilator function, suggesting an involvement of CYP-derived metabolites in EDH-mediated vasodilator responses (Zhao et al., 2005). However, in the relaxation induced by G-1 in SHR mesenteric arteries, the cytochrome P450-mediated response does not appear to participate.

According to Matoba and Shimokawa (2003), in arterial resistance segments H_2_O_2_ is considered an EDH. Therefore, to verify its participation we carried out conjugated incubations with L-NAME plus INDO and catalase, an enzyme that decomposes H_2_O_2_. The vasodilator response was impaired after incubation with catalase in both sexes, although females suffered a greater impairment, indicating that the EDH pathway seems to be more important in females than in males. Indeed, Wong et al. (2015) described sex differences related to the endothelium-dependent relaxation pathway in microvessels of pig coronary arteries, with males having exhibited greater dependence on the NO pathway and less participation of the EDH pathway, unlike females, in whom the EDH pathway was predominant. This lower dependence on the EDH pathway was explained by males showing higher oxidative stress than females. In addition, increased activity and expression of NOX4 has been described in females, what was associated with the production of H_2_O_2_ (Ray et al., 2011).

When arterial segments were incubated with catalase alone, males showed an increase in maximum relaxation response as well as in AUC. In females, the response remained unchanged. H_2_O_2_ has an important role in the cross-talk between NOS and COX: In low concentrations, H_2_O_2_ increases NOS activity, while in high concentrations it increases mainly the endothelial COX activity (Silva et al., 2017). SHR vessels have characteristic endothelial dysfunction not only due to decreased EDRF release, but also as a result of the simultaneous release of endothelium-derived contracting factors (EDCFs). Indeed, the study by Luscher and Vanhoutte (1986) showed that indomethacin restores SHR aorta relaxation to normotensive levels, thus suggesting that these EDCFs should be products of COX in males. In SHR there is an increase in the production of contractile PNs by COX (Félétou et al., 2009). The release of EDCFs is exacerbated in hypertension, and selective COX inhibitors abolish endothelium-dependent contraction in the SHR aorta (Tang and Vanhoutte, 2009; Silva et al., 2012). In addition, PGI_2_ induces vasodilation in a physiological context, whereas in elderly animals or in SHR it induces contraction (Vanhoutte, 2011). In this study, the increase in the relaxation response induced by G-1 after catalase incubation may be related to the reduction of COX activity due to the reduction of H_2_O_2_ and, consequently, a reduction in the formation of vasoconstrictor PNs. These results are similar to those obtained upon incubation with indomethacin. We did not find the same outcome in females, probably because estrogen can modulate the production of vasoconstrictor PNs, thus protecting female SHR against hypertension by decreasing the synthesis of EDCFs such as PGH_2_/PGF_2_α (Dantas et al., 1999).

Our fluorescence results demonstrated that the activation of GPER is capable of increasing the fluorescence intensity of H_2_O_2_ in males and females, although it did not increase of ROS. Peixoto et al. (2018) also found no increase in ROS after stimulation with G-1 in mesenteric resistance arteries of normotensive rats of both sexes. Broughton et al. (2010) found that G-1 was able to reduce ROS formation in carotid and intracranial cerebral arteries. On the other hand, Debortoli et al. (2017) observed an increase in ROS in coronary arteries of male normotensive rats, and Yao and Abdel-Rahman (2016) showed that GPER blockade attenuated ethanol- evoked increases in ROS. All in all, the effect of GPER on oxidative stress is not fully elucidated. Meyer et al. (2016) demonstrated that the genetic ablation of GPER is able to reduce oxidative stress, thus preventing angiotensin II-induced hypertension. Meyer and Barton (2018) suggested the use of the specific GPER antagonist, G36, as a new therapy for cardiovascular diseases, due to its ability to reduce the formation of ROS through downregulation of NOX1.

Taken together, our results demonstrated that GPER activation promotes a relaxing response in resistance mesenteric arteries of hypertensive rats without sex differences, but with the participation of different endothelial mediators. Males appear to be more dependent on the NO pathway, followed by the H_2_O_2_ pathway, and females on the endothelium and H_2_O_2_ pathway. Within this context, it is clear that the redox state of endothelial cells, as well as their interaction with VSM, are determining factors for the type of mechanism involved in the relaxation response. Thus, the role of GPER in vascular function associated with oxidative stress is not yet fully understood. These results are important to the understanding of GPER activation, as well as of estrogenic actions in the vascular system associated with hypertension, the most prevalent disease in the world.

## Data Availability Statement

The raw data supporting the conclusions of this article will be made available by the authors, without undue reservation.

## Ethics Statement

The animal study was reviewed and approved by the Ethics Committee of the Federal University of Espirito Santo.

## Author Contributions

ND, WR, and RS participated in the study design, wrote first draft of the manuscript, and conducted the data interpretation and analyses. ND, WR, LF-L, and IV-A performed the experiments. VL and RS reviewed the manuscript submitted for publication. All authors revised and approved the final version of the manuscript.

## Conflict of Interest

The authors declare that the research was conducted in the absence of any commercial or financial relationships that could be construed as a potential conflict of interest.

## Reagent List

1-[4-(6-bru6omobenzo [1,3]dioxol-5yl)-3a,4,5,9b-tetrahydro-3Hcyclopenta-[c]quinolin-8-yl]-ethanone (G-1); 2′, 7′ dichlorodihydrofluorescein diacetate fluorescent probe (H2DCF-DA) and dihydroethidium probe were purchased from Cayman Chemical, MI, United States. Phenylephrine (PE), acetylcholine (ACh), N^ω^-nitro-L-arginine methyl ester (L-NAME), indomethacin (INDO), clotrimazole (CLOT), catalase and tiron were purchased from Sigma, MO, United States).

## Publisher’s Note

All claims expressed in this article are solely those of the authors and do not necessarily represent those of their affiliated organizations, or those of the publisher, the editors and the reviewers. Any product that may be evaluated in this article, or claim that may be made by its manufacturer, is not guaranteed or endorsed by the publisher.
